# SARS-CoV-2 Vaccine Uptake during Pregnancy in Regione Lombardia, Italy: A Population-Based Study of 122,942 Pregnant Women

**DOI:** 10.3390/vaccines10081369

**Published:** 2022-08-22

**Authors:** Irene Cetin, Maria Mandalari, Elena Cesari, Catia Rosanna Borriello, Michele Ercolanoni, Giuseppe Preziosi

**Affiliations:** 1 Department of Obstetrics and Gynecology, Università degli Studi di Milano, Vittore Buzzi Children’s Hospital, 20154 Milano, Lombardia, Italy; 2 Vittore Buzzi Children’s Hospital, 20154 Milano, Lombardia, Italy; 3 Directorate General for Health, 20124 Milano, Lombardia, Italy; 4 ARIA S.p.A. (The Innovation and Procurement Regional Company of Regione Lombardia), 20124 Milano, Lombardia, Italy

**Keywords:** SARS-CoV-2, vaccination, pregnancy, health policy

## Abstract

Italy has been one of the hardest hit countries in the European Union since the beginning of the SARS-CoV-2 pandemic, and Regione Lombardia (RL) has reported the largest number of cases in the country. This population-based retrospective study analyzed RL records of 122,942 pregnant women to describe SARS-CoV-2 vaccination uptake in the pregnant population, to compare pregnant women vaccine uptake vs. women of childbearing age and to evaluate the impact of vaccination status in pregnant women on admissions to intensive care units during 2021. Vaccination uptake according to citizenship and educational level and the comparison between pregnant and non-pregnant women was performed by Z test. A logistic regression was performed to compare age groups. Out of 122,942 pregnant women, 79.9% were vaccinated at the end of 2021. The vaccine uptake rate was significantly lower in pregnant versus non-pregnant women but increased after the issuing of official recommendations. Vaccine administration was significantly higher among pregnant women with Italian citizenship and with a high level of education in all trimesters. In conclusion, the role of official recommendations with explicit communication about the importance and safety of vaccination in pregnancy is critical to obtain trust and acceptance among pregnant women.

## 1. Introduction

When the first SARS-CoV-2 vaccines became available, evidence to offer vaccination in pregnancy was limited as pregnant women were excluded from pre-marketing clinical trials [1]. The first issued recommendations, supported by the main international associations, only indicated SARS-CoV-2 vaccination for breastfeeding mothers and pregnant women at higher risk of exposure to the virus or at greater risk of developing a severe illness [2,3,4,5,6,7]. On 8 January 2021, the WHO confirmed the safety and efficacy of mRNA vaccines, reiterating that at the time of publication, no evidence was available about safety and efficacy for women in pregnancy and lactation [8]. The first report of the “v-safe” register, established in the US by the CDC for the surveillance of women vaccinated during all trimesters of pregnancy (published on 21 April 2021), showed no concerns regarding the safety profile of SARS-CoV-2 mRNA vaccines in pregnant women [9].

At the same time, studies demonstrated a higher risk of severe COVID-19 disease and adverse pregnancy outcome in pregnant compared to non-pregnant women [10,11,12], with higher ICU admissions compared to non-pregnant SARS-CoV-2-infected women [13]. Among the most critically ill pregnant and postpartum women, almost none were vaccinated [14].

This knowledge supported the rapid development of recommendations for SARS-CoV-2 vaccination for all pregnant and breastfeeding women and those trying to conceive [15] Nevertheless, the cumulative SARS-CoV-2 vaccine acceptance rate among pregnant women appeared low [16].

The primary aim of this study was to evaluate the SARS-CoV-2 vaccination uptake in the whole pregnant population of Regione Lombardia (RL). Secondary outcomes were the comparison between the pregnant and the non-pregnant female population of childbearing age and the impact of vaccination status in pregnant women on admissions to intensive care units during 2021. We also evaluated SARS-CoV-2 vaccination uptake in the pregnant population according to sociodemographic characteristics.

## 2. Materials & Methods

Population. All pregnancies occurring between 1 January 2021 and 31 December 2021 in RL were enrolled in this study. Pregnant women were identified through specific obstetric codes from all medical referrals of the study period; the gestational week reported on the medical records was utilized to calculate an average date of conception (2 weeks after the first day of the last menstrual period).

Information on gestation length, educational level, age, citizenship and admission to ICU were obtained from two databases of Regione Lombardia, CedAP and SDO. Briefly, CedAP is a “birth assistance certificate” collected by a midwife at each delivery with basic sociodemographic information and data on pregnancy outcome. SDO is the system collecting all “Hospital Discharge Records”, providing information relating to each patient discharged from all public and private medical facilities throughout the Italian national territory. Data obtained are pseudonymized through the irreversible encryption algorithms of the SHA256 HMAC 256 BIT type, in compliance with the legislation on the protection of personal data.

In order to calculate the end of pregnancy, we utilized data obtained from crossing the data with the CedAP database to obtain the week of the end of the gestation for those pregnancies that delivered in 2021.

CedAP was also used to derive the educational level of pregnant women. This information was only available for pregnant women with at least one completed CedAP, i.e., pregnant women who gave birth in 2021 or with a CedAP already completed at previous deliveries (*n* = 95,273). The educational level was considered high for women with a university degree or more and low for women with primary school or no qualifications, middle school or high school.

SDO records were utilized for date of birth, citizenship and information about admission to intensive care units.

Vaccination in pregnancy was defined as vaccination given at any point from the date of conception to the date the pregnancy ended included. Two types of mRNA vaccines were utilized during pregnancy in RL (Pfizer-BioNTech BNT162b2 messenger RNA vaccine and Moderna mRNA-1273 mRNA vaccine).

Vaccine uptake was also analyzed in the general non-pregnant female population of childbearing age (women between 15 and 49 years). Data were obtained from databases of RL.

### 2.1. Policy for Vaccine Distribution in Regione Lombardia in the Study Period

**Figure 1:**
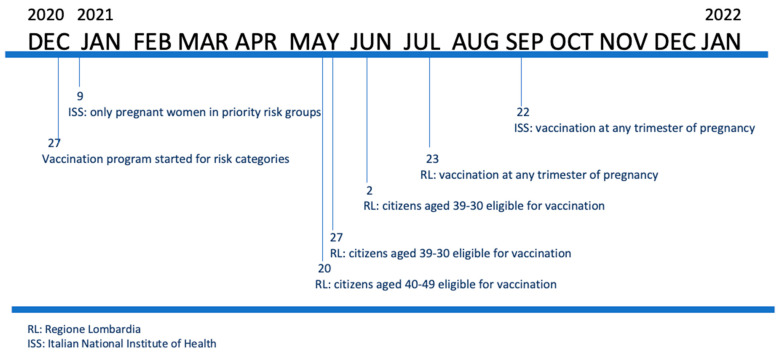
Pregnant population invited for COVID-19 vaccination in RL over time and timeline of Italian and regional guidelines for vaccination against SARS-CoV-2 in pregnant women.

SARS-CoV-2 vaccination became available in RL in the last days of 2020 (27 December). Figure 1 describes the progressive population categories that were eligible for vaccination in RL.

In agreement with the National Plan, RL organized the vaccination campaign in two subsequent steps: in the first phase, priority for SARS-CoV-2 vaccination was given to healthcare professionals (HCPs), residents in nursing homes, elderly people older than 80 years and highly vulnerable patients, independent of their age. Secondly, other categories with different risk levels were identified according to an age criterion from 75 to 79 years at first and afterwards people belonging to lower age categories. Therefore, women of childbearing age started to be vaccinated on 20 May 2021 (age, 40–49), and then on 27 May, the vaccine was provided to citizens aged 30–39 and on 2 June 2021 to citizens aged 12–29.

### 2.2. Vaccine Recommendations during Pregnancy

Figure 1 also presents the timing of guidelines and policies for vaccination against SARS-CoV2 in pregnant women. In the first weeks after the vaccines became available, no specific Italian recommendations indicated pregnancy as a priority class, since pregnant women had not been included in the clinical trials of mRNA vaccines.

On 9 January 2021, the Italian Obstetric Surveillance System (ItOSS) published indications about vaccination against SARS-CoV-2 in pregnancy: routine vaccination in pregnant women was not recommended, and vaccination was only considered for pregnant women belonging to risk categories such as HCPs or caregivers and/or at risk of serious complications from COVID 19.

After 6 months, a panel of experts in RL provided specific indications for the HCPs of the region, dated 23 July 2021, that recommended vaccination for all pregnant women in any trimester of pregnancy.

Then, on 22 September 2021, the ItOSS posted an update that indicated vaccination at any stage of pregnancy, with a note of caution for the first trimester, justified in that document by too little evidence of vaccinations performed in the first trimester.

### 2.3. Patient and Public Involvement

We utilized routinely generated anonymized regional data obtained from hospital records and from COVID-19 vaccine databases. No patients were involved in setting the study question, nor were they involved in developing plans for the design or implementation of the research. No patients were involved in the interpretation or writing up of the results.

### 2.4. Statistical Analysis

Data about SARS-CoV-2 vaccine uptake in pregnant women and in non-pregnant women of childbearing age are presented as numbers and frequency (%). The comparison between the group of pregnant and non-pregnant women was carried out at the end of three representative months (31 July 2021, 30 September 2021 and 31 December 2021) by Z test. Z test was also performed to compare vaccine uptake according to citizenship and educational level. A logistic regression was performed to compare age groups. Findings were reported as significant if *p* < 0.05. All the data were analyzed through SAS software.

## 3. Results

**Figure 2:**
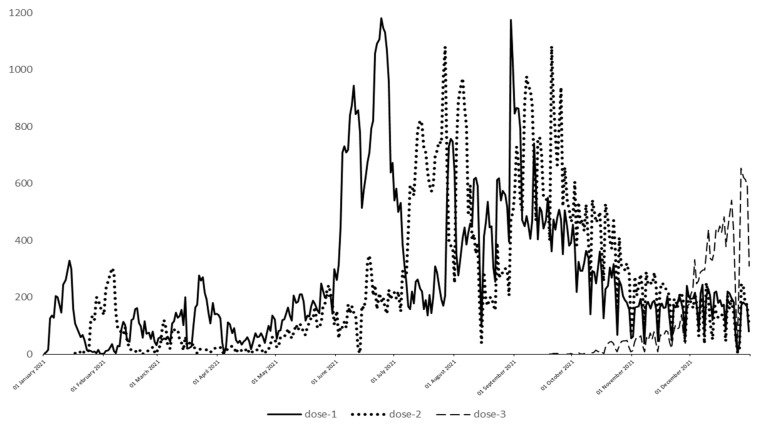
SARS-CoV-2 vaccine uptake among pregnant women in RL of I, II and III doses.

Overall, records indicated that 122,942 women were pregnant in 2021 in RL. Figure 2 describes the uptake of SARS-CoV-2 vaccination in the pregnant population throughout the year for I, II and III doses. SARS-CoV-2 vaccine coverage increased over the year with 98,219 women receiving the first dose during pregnancy by the end of December 2021 (79.9%). Table 1 presents the characteristics of the full cohort divided considering sociodemographic characteristics of the general pregnant population. Out of 122,942 women that were identified as pregnant in 2021 (aged 15–49), 65,688 gave birth in 2021, while 8175 pregnancies ended prematurely (before the stage of neonatal viability identified at 22 gestational weeks).

### 3.1. SARS-CoV-2 Vaccine Coverage in the Pregnant Compared to the Non-Pregnant Population

**Figure 3:**
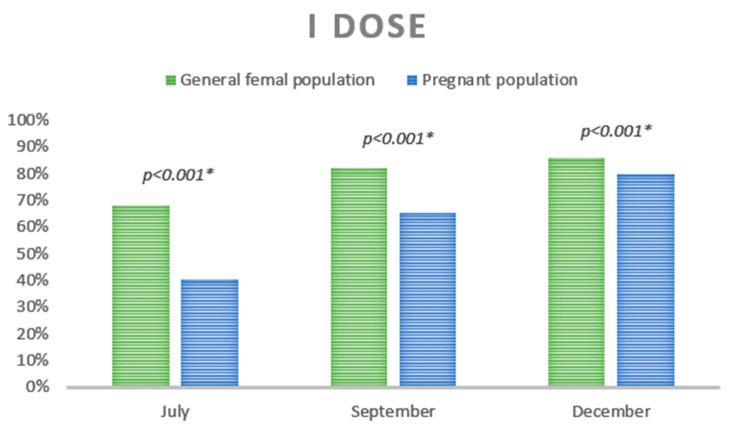
SARS-CoV-2 vaccine uptake of I dose in the general female population (15–49 yrs) vs. the pregnant population. * Z Test.

The comparison of SARS-CoV-2 vaccine uptake in women who were pregnant in 2021 versus non-pregnant women aged 15–49 in the same time period is presented in Figure 3.

Particularly, the end of the three time periods was evaluated: July (31 July 2021), September (30 September 2021) and December (31 December 2021). As shown in Figure 1, these periods correspond to the publication of specific new indications about the population eligible for vaccination. In all the three months analyzed, the percentage of non-pregnant women vaccinated with the I dose was significantly higher (July: 68.3%, September: 82.3% and December: 86.1%) than that of the pregnant population (July: 40.4%, September: 65.4% and December: 79.9%) (*p* < 0.001).

### 3.2. Impact of Vaccination Status on SARS-CoV-2-Related Admission to ICU during Pregnancy

During 2021, 53 pregnant women with a COVID-19 diagnosis were admitted to ICU in Regione Lombardia. All these 53 women were unvaccinated, i.e., they had not received any dose of vaccine against SARS-CoV-2 either during or before pregnancy. Overall, these women represented 0.04% of the total number of women that were pregnant in RL during 2021 (53 out of 122,942).

### 3.3. SARS-CoV-2 Vaccine Coverage and Uptake in Pregnancy

Data about vaccine coverage and uptake of the I dose in the three trimesters of pregnancy are presented in Table 2 and Table 3 according to sociodemographic characteristics.

The results show that Italian pregnant women were significantly more vaccinated than foreign pregnant women in all three trimesters of pregnancy. Vaccine uptake was also analyzed in relation to three age categories (<30 years, between 30 and 39 years and ≥40 years) (Table 2). A significantly higher number of pregnant women aged ≥ 40 were vaccinated in all trimesters of pregnancy, followed by pregnant women aged 30–39 (Table 2).

Table 3 shows SARS-CoV-2 vaccine uptake in the pregnant population according to educational level. Vaccine uptake was significantly higher in pregnant women with a high compared to pregnant women with a lower educational level in every trimester (*p* < 0.001).

## 4. Discussion

Here we present data about vaccination uptake against SARS-CoV-2 in the pregnant population of RL. At the end of 2021, 98,219 women were vaccinated with at least one dose during pregnancy, representing 79.9% of women that were pregnant during 2021. To our knowledge, this is the largest reported population-based cohort of pregnant women vaccinated against SARS-CoV-2 and the only one conducted in Italy.

Only few studies about pregnant women adherence to SARS-CoV-2 vaccination have been published thus far, showing differences worldwide, likely in relation to the timing of the pandemic development and to local guidelines. The uptake during pregnancy has been greater in countries firstly significantly affected by the SARS-CoV-2 infection, such as China, where the acceptance rate of a COVID-19 vaccine in pregnant women was as high as 77.4% [17] and similar to our finding of 79.9%. In other countries, such as United Kingdom, the SARS-CoV-2 vaccination was accepted only by 28.5% of eligible pregnant women, but this study was conducted in the period between 1 March 2020 and 4 July 2021 [18]. Our data are similar as the SARS-CoV-2 vaccine uptake in the pregnant population of Regione Lombardia was low in the first half of 2021, as shown in Figure 2. Conversely, it increased from June 2021. The authors stated that changes in the United Kingdom guidance along with the lack of safety and efficacy data likely contributed to vaccine hesitancy among pregnant women. A similar study conducted by Stock et al. between 8 December 2020 and 31 October 2021, showed a higher rate of vaccination in the British pregnant population but still lower than the rate of vaccination in the general female population; this result reflects the same trend reported by our study [10].

Our data also show how vaccination against SARS-CoV-2 during pregnancy increased over time. Changes in the level of acceptance among pregnant women may be related to both logistical and knowledge factors. Indeed, a first, progressive increase in vaccine uptake was observed from May 2021, when the SARS-CoV-2 vaccine became available for the age category of women of childbearing age. A second, consistent increase observed from the end of July is explained by the rising knowledge about SARS-CoV-2 vaccine safety followed by the publication of official recommendations on maternal vaccination against SARS-CoV-2.

Unlike other vaccines previously utilized in pregnancy, the SARS-CoV-2 vaccines used for pregnant women were the new mRNA vaccines, considered a priori relatively safe for pregnant women given the absence of live or attenuated virus. The vaccine was also shown to be unable to cause genetic changes, as it does not enter the nucleus of the cells, while the mRNA naturally degrades after a few days [19].

The role of official recommendations by institutions was indeed critical, as indicated by the considerable increase in vaccination of pregnant women observed after the recommendations were issued. A first increase was evident after the publication of recommendations by RL at the end of July followed by a second peak in September after the ISS issued indications for all pregnant women.

In the same time period, increasing evidence of higher risk of severe COVID-19 infection in pregnant women rather than in the general population was published [11]. This, together with data indicating trans placental transfer of maternal antibodies after SARS-CoV-2 vaccination during the third trimester [20,21], which suggests that maternal vaccination might provide some level of protection to the neonate, likely also led to increasing adherence to SARS-CoV-2 vaccination among pregnant women.

Vaccine hesitancy has been reported in pregnancy, with barriers to vaccination mainly depending on low knowledge about safety, need or effectiveness of vaccination and lack of recommendation by an HCP [22]. Instead, the main factors identified to increase the acceptance of SARS-CoV-2 vaccination in pregnancy were explicit communication about the safety of SARS-CoV-2 vaccines for pregnant women, trust in the importance and effectiveness of the vaccine and in the health system, belief in the importance and acceptance of other vaccinations in pregnancy, such as those for influenza or pertussis, together with anxiety about COVID-19 [23,24].

We previously reported significantly lower percentages of vaccine uptake for influenza and pertussis in Italian pregnant women (approximately 15% and 60%, respectively) than what we currently report for SARS-CoV-2 vaccination. The main vaccination barriers previously identified were lack of vaccine recommendation by any HCP and safety concerns [25], while the greatest motivation was protection of the baby [26]. Concerns about the health of the mother and the baby as well as massive information and recommendations might explain the difference.

Data were also analyzed using three sociodemographic characteristics: age, citizenship and education; as in previous studies, older age, higher education and white ethnicity have been associated with higher acceptance of the vaccine [24,27]. Similarly, in our population, the highest percentage of vaccinated pregnant women was recorded in Italian pregnant women with a high level of education and with older age.

Although we report a high rate of vaccination uptake, our data also show that this rate was consistently and significantly lower among pregnant women than in the general female population of childbearing age. The difference decreased progressively during the year, as official indications regarding vaccination in pregnancy were published. These recommendations were driven by the evidence that pregnant women were at high risk of developing severe disease [11].

A recent study combining data from six countries within the International Network of Obstetric Survey Systems also indicated that unvaccinated pregnant women are more likely to be admitted to ICU than vaccinated pregnant women [28]. Our data confirm this finding since none of the 53 pregnant women admitted to ICU in RL in 2021 were vaccinated.

### Strengths and Limitations

A major strength of this study is the large epidemiological sample of pregnant patients representing the whole population of Regione Lombardia during 2021. The study utilized different databases representative of the whole pregnant and non-pregnant population providing data about vaccine uptake in all trimesters of pregnancy and according to sociodemographic characteristics in a highly impacted region. A limitation concerns the data about the educational level that could be retrieved only from the CedAP database, therefore only for those pregnancies that delivered after 22 weeks. A further potential limitation is the regional origin that may limit the applicability to other populations.

## 5. Conclusions

SARS-CoV-2 vaccination uptake increased progressively among pregnant women in RL with the progressive availability of the vaccine and the issuing of official recommendations, but was always lower than in the female non-pregnant population of childbearing age. SARS-CoV-2 vaccination in pregnant women seems to prevent severe COVID-19 disease and admission to intensive care unit. The acceptance of SARS-CoV-2 vaccination was greater among pregnant women with Italian citizenship and a higher level of education and increased with maternal age.

The role of official recommendations with explicit communication about the importance and safety of vaccination in pregnancy is critical to obtain trust and acceptance among pregnant women. Accurate patient counseling on vaccination risks and benefits during pregnancy and maternal vaccination strategies that consider specific perspectives and characteristics of this population are necessary in order to reach and protect the majority of pregnant women.

## Figures and Tables

**Table 1:** Sociodemographic characteristics of the pregnant population.

<30		30–39	≥40
Age (yrs)	31,419 (25.6%)	77,030 (62.6%)	14,493 (11.8%)
	Italian	Foreigners	
Citizenship	88,994 (72.4%)	33,948 (27.6%)	
	Low level	High level	
Education	58,853 (61.8%)	36,420 (38.2%)	

**Table 2:** SARS-CoV-2 vaccine I dose uptake in pregnant population according to citizenship and age in the three trimesters of pregnancy.

	Citizenship			Age (yrs)					
	Italian n = 88,994	Foreigners n = 33,948	*p* *	<30 yrs n = 31,419	30–39 yrs n =77,030	≥40 yrs n = 14,493	*p* ** (<30 yrs vs. ≥40 yrs)	*p* ** (<30 yrs vs. 30–39 yrs)	*p* ** (≥40 yrs vs 30–39 yrs)
Vaccinated I trimester	8328 (9.4%)	697 (2%)	<0.001	1554 (4.9%)	6193 (8%)	1278 (8.8%)	<0.001	<0.001	<0.001
Vaccinated II trimester	34,250 (38.5%)	5511 (16.2%)	<0.001	6004 (19.1%)	27,551 (35.8%)	6206 (42.8%)	<0.001	<0.001	<0.001
Vaccinated III trimester	62,997 (70.8%)	17,476 (51.5%)	<0.001	17,701 (56.3%)	52,271 (67.9%)	10,501 (72.5%)	<0.001	<0.001	<0.001

* Z test, ** Logistic regression. Data collected through SDO database (*n* = 122,942 pregnant women); data presented as number and (%).

**Table 3:** SARS-CoV-2 vaccine I dose uptake in pregnant population according to educational level in the three trimesters of pregnancy.

Education	Low *n* = 58,853	High *n* = 36,420	*p* *
Vaccinated I trimester	1458 (2.5%)	3973 (10.9%)	<0.001
Vaccinated II trimester	12,708 (21.6%)	13,617 (37.4%)	<0.001
Vaccinated III trimester	33,560 (57.0%)	26,321 (72.3%)	<0.001

* Z test. Data collected through CedAP database (*n* = 95,273); data presented as number and (%).

## Data Availability

The data that support the findings of this study are available from Regione Lombardia Health Service, but restrictions apply to the availability of these data, which were used under license for the current study, and so are not publicly available. Data are, however, available from Regione Lombardia, Directorate General for Health, upon reasonable request.

## References

[1] Luxi N., Giovanazzi A., Capuano A., Crisafulli S., Cutroneo P.M., Fantini M.P., Ferrajolo C., Moretti U., Poluzzi E., Raschi E. (2021). COVID-19 vaccination in pregnancy, paediatrics, immunocompromised patients, and persons with history of allergy or prior SARS-CoV-2 infection: Overview of current recommendations and pre- and post-marketing evidence for vaccine efficacy and safety. Drug Saf..

[2] European Medicine Agency EMA Recommends First COVID-19 Vaccine for Authorisation in the EU. https://www.ema.europa.eu/en/news/ema-recommends-first-covid-19-vaccine-authorisation-eu.

[3] Centers for Disease Control and Prevention Vaccine Recommendations and Guidelines of the Advisory Committee on Immunization Practices (ACIP) Interim Considerations for COVID-19 Vaccination of Healthcare Personnel and Long-Term Care Facility Residents. https://www.cdc.gov/vaccines/hcp/acip-recs/vacc-specific/covid-19/clinical-considerations.html.

[4] American College of Obstetricians and Gynecologists Vaccinating Pregnant and Lactating Patients against COVID-19. https://www.acog.org/clinical/clinical-guidance/practiceadvisory/articles/2020/12/vaccinating-pregnant-and-lactating-patients-against-covid-19.

[5] Pfizer-BioNTech COVID-19 Vaccine (CoVID-19 mRNA Vaccine) Product Monograph (Canada). https://covid-vaccine.canada.ca/info/pdf/pfizer-biontech-covid-19-vaccine-pm1-en.pdf.

[6] The Society of Obstetricians and Gynecologists of Canada Statement on COVID-19 Vaccination in Pregnancy. https://sogc.org/common/Uploaded%20files/Latest%20News/SOCG_Statement_COVID19_Vaccination_in_Pregnancy.pdf.

[7] Medicines & Health Care Products Regulatory Agency Regulatory Approval of Pfizer/BioNTech Vaccine for COVID-19. Information for Healthcare Professionals on Pfizer/BioNTech COVID-19 Vaccine. https://www.gov.uk/government/publications/regulatory-approval-of-pfizerbiontech-vaccine-for-covid-19.

[8] Zambrano L.D., Ellington S., Strid P., Galang R.R., Oduyebo T., Tong V.T., Woodworth K.R., Nahabedian J.F., Azziz-Baumgartner E., Gilboa S.M. (2020). Update: Characteristics of symptomatic women of reproductive age with laboratory-confirmed Sars-CoV-2 infection by pregnancy status—United States, 22 January–3 October 2020. Morb. Mortal. Wkly. Rep..

[9] Shimabukuro T.T., Kim S.Y., Myers T.R., Moro P.L., Oduyebo T., Panagiotakopoulos L., Marquez P.L., Olson C.K., Liu R., Chang K.T. (2021). Preliminary Findings of mRNA COVID-19 Vaccine Safety in Pregnant Persons. N. Engl. J. Med..

[10] Stock S.J., Carruthers J., Calvert C., Denny C., Donaghy J., Goulding A., Hopcroft L.E.M., Hopkins L., McLaughlin T., Pan J. (2022). SARS-CoV-2 infection and COVID-19 vaccination rates in pregnant women in Scotland. Nat. Med..

[11] Villar J., Ariff S., Gunier R.B., Thiruvengadam R., Rauch S., Kholin A., Roggero P., Prefumo F., Vale M.S.D., Cardona-Perez J.A. (2021). Maternal and neonatal morbidity and mortality among pregnant women with and without COVID-19 infection: The INTERCOVID Multinational Cohort Study. JAMA Pediatr..

[12] Allotey J., Stallings E., Bonet M., Yap M., Chatterjee S., Kew T., Zhou D., Coomar D., Sheikh J., Lawson H. (2020). Clinical manifestations, risk factors, and maternal and perinatal outcomes of coronavirus disease 2019 in pregnancy: Living systematic review and meta-analysis. BMJ.

[13] Khan D.S.A., Pirzada A.N., Ali A., Salam R.A., Das J.K., Lassi Z.S. (2021). The Differences in Clinical Presentation, Management, and Prognosis of Laboratory-Confirmed COVID-19 between Pregnant and Non-Pregnant. Int. J. Environ. Res. Public Health.

[14] Engjom H., Akker T.V.D., Aabakke A., Ayras O., Bloemenkamp K., Donati S., Cereda D., Overtoom E., Knight M. (2022). Severe COVID-19 in pregnancy is almost exclusively limited to unvaccinated women—time for policies to change. Lancet Reg. Health Eur..

[15] Centers for Disease Control and Prevention Pregnant and Recently Pregnant People at Increased Risk for Severe Illness from COVID-19. https://www.cdc.gov/coronavirus/2019-ncov/need-extra-precautions/pregnant-people.html#anchor_1623351182596.

[16] Carbone L., Di Girolamo R., Mappa I., Saccone G., Raffone A., Di Mascio D., De Vivo V., D’Antonio F., Guida M., Rizzo G. (2022). Worldwide beliefs among pregnant women on SARS-CoV-2 vaccine: A systematic review. Eur. J. Obs. Gynecol. Reprod. Biol..

[17] Tao L., Wang R., Han N., Liu J., Yuan C., Deng L., Han C., Sun F., Liu M., Liu J. (2021). Acceptance of a COVID-19 vaccine and associated factors among pregnant women in China: A multi-center cross-sectional study based on health belief model. Hum. Vaccin. Immunother..

[18] Blakeway H., Prasad S., Kalafat E., Heath P.T., Ladhani S.N., Le Doare K., Magee L.A., O’Brien P., Rezvani A., von Dadelszen P. (2022). COVID-19 vaccination during pregnancy: Coverage and safety. Am. J. Obs. Gynecol..

[19] Centers for Disease Control and Prevention Understanding mRNA COVID-19 Vaccines. https://www-cdc-gov.pros2.lib.unimi.it/coronavirus/2019-ncov/vaccines/different-vaccines/mrna.html.

[20] Gill L., Jones C.W. (2021). Severe Acute Respiratory Syndrome Coronavirus 2 (SARS-CoV-2) Antibodies in Neonatal Cord Blood After Vaccination in Pregnancy. Obs. Gynecol..

[21] Gray K.J., Bordt E.A., Atyeo C., Deriso E., Akinwunmi B., Young N., Baez A.M., Shook L.L., Cvrk D., James K. (2021). Coronavirus disease 2019 vaccine response in pregnant and lactating women: A cohort study. Am. J. Obstet. Gynecol..

[22] Wilson R.J., Paterson P., Jarrett C., Larson H.J. (2015). Understanding factors influencing vaccination acceptance during pregnancy globally: A literature review. Vaccine.

[23] Januszek S.M., Faryniak-Zuzak A., Barnaś E., Łoziński T., Góra T., Siwiec N., Szczerba P., Januszek R., Kluz T. (2021). The approach of pregnant women to vaccination based on a COVID-19 Systematic Review. Medicina.

[24] Skjefte M., Ngirbabul M., Akeju O., Escudero D., Hernandez-Diaz S., Wyszynski D.F., Wu J.W. (2021). COVID-19 vaccine acceptance among pregnant women and mothers of young children: Results of a survey in 16 countries. Eur. J. Epidemiol..

[25] Vilca L.M., Sarno L., Cesari E., Vidiri A., Antonazzo P., Ravennati F., Cavaliere A.F., Guida M., Cetin I. (2021). Differences between influenza and pertussis vaccination uptake in pregnancy: A multi-center survey study in Italy. Eur. J. Public Health.

[26] Maisa A., Milligan S., Quinn A., Boulter D., Johnston J., Treanor C., Bradley D.T. (2018). Vaccination against pertussis and influenza in pregnancy: A qualitative study of barriers and facilitators. Public Health.

[27] Levy A.T., Singh S., Riley L.E., Prabhu M. (2021). Acceptance of COVID-19 vaccination in pregnancy: A survey study. Am. J. Obstet. Gynecol. MFM.

[28] Donati S., Corsi E., Maraschini A., Salvatore M.A., ItOSS-COVID-19 Working Group (2022). SARS-CoV-2 infection among hospitalized pregnant women and impact of different viral strains on COVID-19 severity in Italy: A national prospective population-based cohort study. BJOG.

